# Case of Bending and Partial Fracture of the Clavicle

**Published:** 1848-08

**Authors:** C. Green

**Affiliations:** Homer, N. Y.


ART. III.—*Case of Bending and Partial Fracture of the Clavicle.* By C. Green, M. D., Homer, N. Y.

*Dec.* 21st. 1847.—Was called to dress, what was supposed to be a fractured clavicle, of George Stone, a lad eight years of age. One of his play-mates had tripped him in such a manner that he fell on his side, striking on the extremity of the left shoulder. I found that he was unable to raise the hand to the head. On examination, I discovered on the posterior edge of ths clavicle, at the inner extremity of the external curvature, a point which was swollen, tender, and painful. The anterior edge of the clavicle was continuous, and there was neither crepitus, nor displacement. Considering the age of the patient, and the appearance of the parts, I diagnosed, bending of the clavicle forward, with a *splitting out* of the posterior edge; and that the bone, by its elasticity, had resumed its ordinary direction. In order to be safe, however, I dressed the shoulder as for actual fracture of the clavicle, lest the fracture *might* have extended nearly through the bone, and there be subsequent displacement. The swelling subsided in 4 or 5 days, and as all seemed secure, I removed the dressings, and heard no more of the matter until the 11th of May, ult., when I was called to see this patient again; and found that he had met, the day before, with precisely the same accident, at the old point, and by the same cause—being tripped down by a playmate. This time, the swelling, and other symptoms of inflammation were greater than before. The anterior edge of the clavicle was entirely continuous, but he could not raise the arm. I merely directed him to keep to his bed until the swelling and inflammation should, in a measure, subside. In three or four days he was about. The callus left is not large, still it is quite evident.

I have had one well marked case of bending of the radius and ulna, in a boy eight years of age, and other surgeons have detailed similar accidents to the fore-arm, and, more rarely, cases of incomplete fracture with bending of the humerus, leg and thigh; but as a like bending of the clavicle is much less common than either of the others, I have thought this case possessed sufficient interest to make it worthy of being reported.

Homer, June 27th, 1848.

